# Penile Injuries in Wild and Domestic Pigs

**DOI:** 10.3390/ani6040025

**Published:** 2016-03-25

**Authors:** Ulrike Weiler, Marie Isernhagen, Volker Stefanski, Mathias Ritzmann, Kevin Kress, Charlotte Hein, Susanne Zöls

**Affiliations:** 1 Institute of Animal Science, Hohenheim University, Schloss Hohenheim 1, 70599 Stuttgart, Germany; volker.stefanski@uni-hohenheim.de (V.S.); Kevin.Kress@uni-hohenheim.de (K.K.); CharlotteHein92@gmx.de (C.H.); 2 Clinic for Swine, Ludwig-Maximilians-University Munich, Sonnenstrasse 16, 85764 Oberschleissheim, Germany; Marie_Isernhagen@web.de (M.I.); schweineklinik@med.vetmed.uni-muenchen.de (M.R.); s.zoels@lmu.de (S.Z.)

**Keywords:** pork production, penile injuries, wild boar (WB), domestic boar (DB)

## Abstract

**Simple Summary:**

Male pigs raised for pork production on experimental and commercial farms were evaluated for scars, fresh wounds and severe injuries of the penis. A high incidence of penile injuries (64.0%–94.9% of the animals/farm) was found in boars but not in barrows (castrated males) with even severe wounds in 5.2% to 9.3% of the boars. A similar evaluation of 15 free-ranging wild boars also revealed a considerable proportion of animals with penile injuries. Thus, penis biting is a highly relevant and severe welfare problem in boars which is not limited to intensive production systems.

**Abstract:**

In boars, sexually motivated mounting can not only cause problems such as lameness, but penile injuries are also reported. The relevance of penis biting in boars is discussed controversially, but reliable data is missing. In the present study, boars (*n* = 385) and barrows (*n* = 85) from experimental farms were therefore evaluated for scars, fresh wounds and severe injuries of the penis. Similarly, 321 boars from 11 farms specializing in pork production with boars, and 15 sexually mature wild boars from the hunting season of 2015/16 were included in the study. In domestic boars, a high incidence of penile injuries was obvious (76.6%–91.3% of animals with scars and/or wounds at experimental farms, 64.0%–94.9% at commercial farms). The number of boars with severe injuries was in a similar range in both groups (5.2% *vs.* 9.3%). At commercial farms, the number of scars but not that of fresh wounds increased per animal with age by 0.3 per week. Moreover, raising boars in mixed groups led to about a 1.5 times higher number of scars than in single-sex groups. In wild boars, a considerable proportion of animals (40%) revealed penile injuries, which were even severe in three animals. We therefore conclude that penis biting is a highly relevant and severe welfare problem in the male pig population, but this phenomenon is not limited to intensive production systems.

## 1. Introduction

In 2011, European pork production stakeholders voluntarily committed themselves to omit surgical castration of boars without anesthesia and pain relief after 2018 due to animal welfare problems with this procedure [1]. Pork production with entire males has been regarded as an animal-friendly alternative. However, there is increasing evidence that specific welfare problems may result in production systems with entire males as behavior is influenced by testicular hormones [2,3]. Thus, it is well known that entire males are more aggressive in order to establish social ranking [4,5]. Fighting may be observed, especially if groups are mixed during transport and lairage [6]. Barrows and gilts also fight to establish a hierarchy, but fighting in boars is more intensive and results in more severe lesions of the carcass [2,3]. This problem may be reduced if stable groups are transported to slaughter and mixing is avoided [3,4]. 

Other problems may result from increasing libido, which leads to a pronounced mounting activity as sexual development proceeds [2]. Sexually oriented mounting was reported to be significantly longer and accompanied by the mounted animal's screaming. Mounting activity in male groups increases the risk of leg weakness and injuries when the mounted animal tries to escape [7]. Mounting activity increases gradually with increasing levels of testicular hormones and is reduced if testicular activity is suppressed, e.g., after immunocastration [8]. 

In boars before puberty or in barrows castrated during the first week of life, the penis is fixed in the preputial sheet by the penile frenulum [9]. With increasing sexual maturity, boars acquire the ability to extrude the penis and the frenulum is disrupted during pelvic thrusting. This natural pubertal behavior may lead to problems when penmates start to bite at the extended penis of a mounting boar [9]. Sexual mounting is therefore not only relevant for problems such as lameness and other leg-related ailments, but can also cause severe damage if penile injuries lead to inflammation and thus to new boar-specific welfare problems. In contrast to other welfare issues, reliable data is scarce, and the extent of penile injuries will remain unknown as long as only standard examinations of the carcasses are carried out. To quantify this problem, the penis has to be removed from the preputial sheet after slaughter and examined for recent and older injuries, such as fresh wounds, scars, signs of inflammation and suppuration. In the present study, such systematic examinations were carried out in entire males and castrated domestic pigs as well as in wild boars obtained during the hunting season of 2015/16. 

## 2. Materials and Methods 

### 2.1. Strategy of the Investigation

The present study was carried out in order to quantify the incidence of penis injuries in male pigs and to get a first impression of possible influences on the phenomenon. In the first step, penis injuries in boars and barrows from the experimental farms were evaluated after slaughter. For all the animals, individual data on growth performance was available. Based on these findings (incidence, lesion type), the evaluation scheme and the sample size (limitation of the number of animals evaluated per farm to 20%–40%) were adapted for a study with entire males from commercial farms. Finally, wild boars harvested during the hunting season of 2015/2016 in southwest Germany were included in order to obtain information about this problem under natural conditions. 

### 2.2. Samples from Boars and Barrows Kept in Experimental Farms

Penises of 385 entire boars and 85 barrows (surgically castrated in the first week of life) from three experimental farms (farrow to finishing) in south Germany were collected at the slaughterhouse. The animals (LR × LW × Pi crossbreed) were housed in fattening units on a fully slatted floor from 30 kg (11–12 weeks of life) until slaughter in single-sex groups. Behavior of the animals was recorded throughout the fattening period and post-mortem examinations of joints and stomach for ulcera were carried out. This part of the study has already been published previously [10,11]. At the slaughter line, penises of boars and barrows were collected together with the preputium, separated from the prepuce, and evaluated according to the scoring system described below. In addition, slaughter weight, age at slaughter and penile injuries for each animal were recorded. The number of animals per pen and the space allowance per animal at the farms were also documented (Table 1).

### 2.3. Samples from Domestic Boars (DB) 

Samples from commercial farms from northwest Germany (nine farms) and the Netherlands (two farms) were collected at a large German slaughter plant. From entire males delivered for slaughter on two consecutive days in July, animals from a total of 11 different farms were selected randomly. All farms were known to have experience with the production of entire male pigs, and each delivered a group of at least 40 boars on that day. From these 11 farms, a total of 1015 boars went to slaughter, from which 321 animals were sampled for further evaluation. For each farm, the average slaughter weight of the animals, the average age at slaughter, the genotype of the boars, the number of animals per pen and information as to whether the animals were all males or mixed groups was recorded. 

During evisceration at the slaughter line, the genital tract was excised as usual and the penis, covered by the preputial sheet and the Diverticulum preputiale together with some tissue surrounding the Ostium preputiale, was removed for further evaluation. After sample collection, the preputium was removed in a section room and the Pars libra penis was evaluated as described below in detail. 

### 2.4. Samples from Wild Boars (WB)

Wild boars were sampled for comparative reasons during the hunting season from late November 2015 until mid-January 2016 in the southwest of Germany. A total of 20 wild boars with a weight between 12 kg and 60 kg after bleeding and evisceration were examined. Five animals were pre-pubertal (12–18 kg, penis not easily removable from the preputial sheet) and had no lesions on the penis at all. They were excluded from the further evaluation.

The remaining 15 animals were classified according to the age of the animal, which was estimated by the dental pattern into the age groups below one year (six animals; 25–37 kg), one to two years (six animals 45–55 kg) and above two years (three animals, 48–60 kg). 

### 2.5. Evaluation of Samples

**Figure 1:**
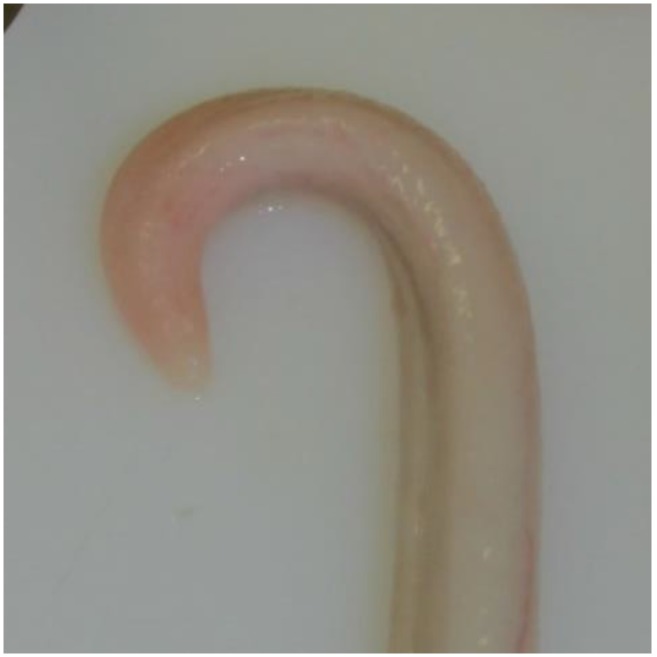
Specimen without injuries (DB).

**Figure 2:**
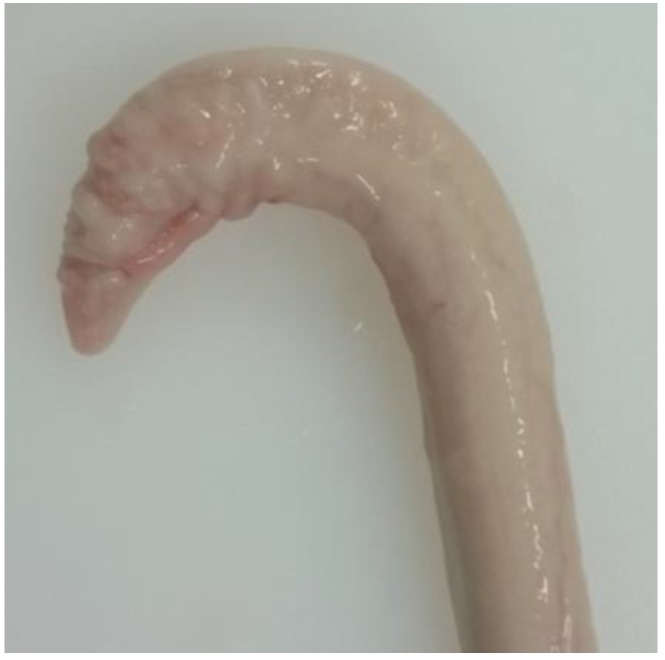
Specimen with multiple scars (DB).

**Figure 3:**
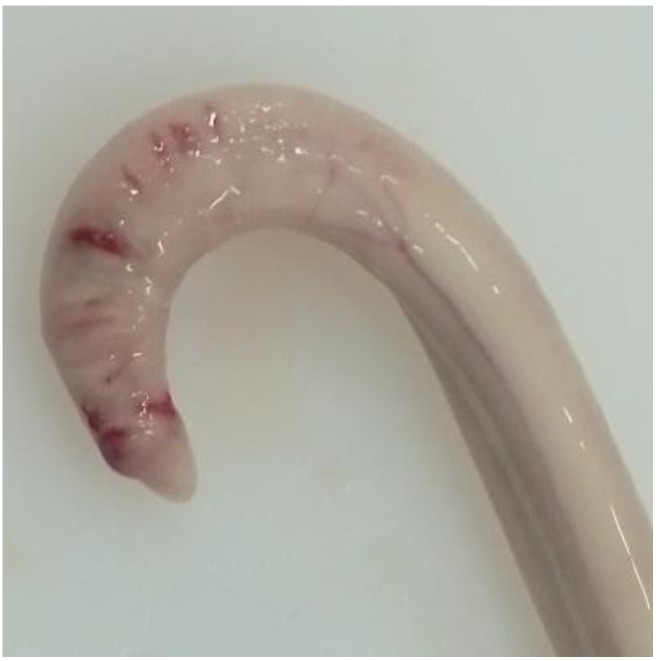
Specimen with fresh injuries (DB).

**Figure 4:**
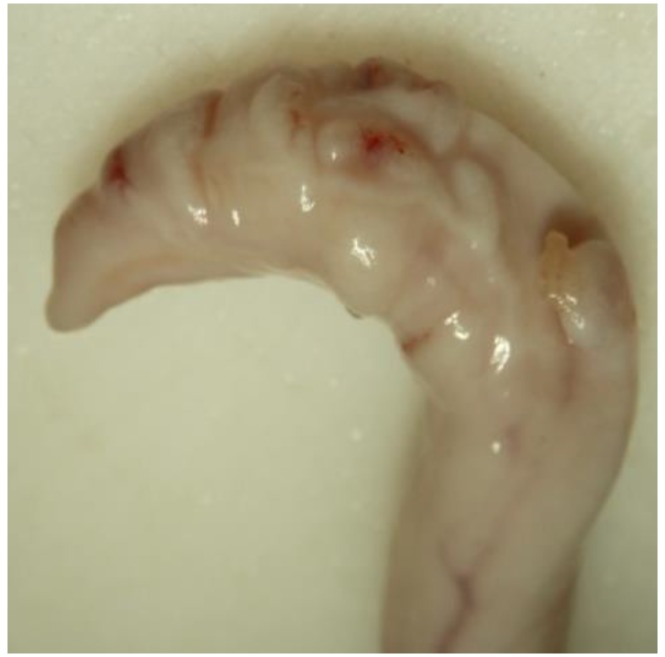
Specimen with suppuration (DB).

**Figure 5:**
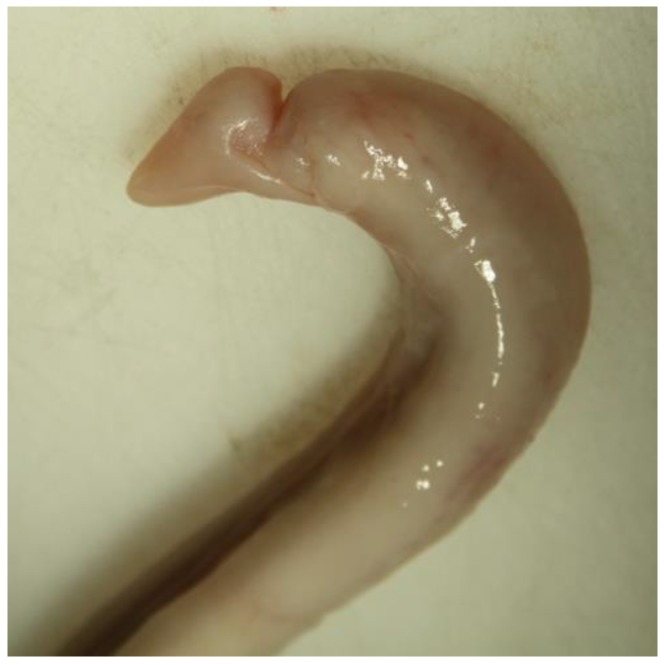
Specimen with partial loss (DB).

**Figure 6:**
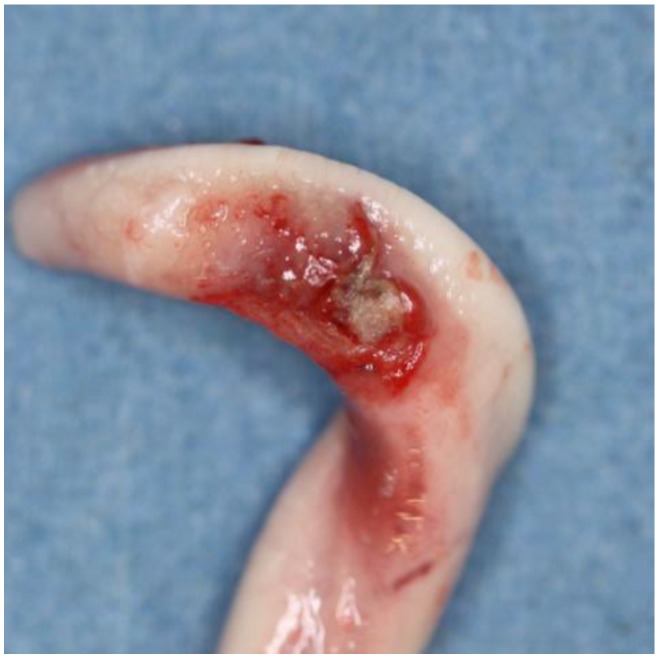
Specimen with severe injury (DB).

Animals with an extruded penis after scalding revealed typical artifacts, especially incinerations due to singeing, and were excluded from the study. In the other animals, the preputium was removed by gentle manual pushing of the penis in a caudal direction within the preputium, so that the preputial sheet could be dissected without affecting the Glans penis or the Pars libra. In pre-pubertal animals or barrows, the adhesion between the preputium and penis via the frenulum was strong and it was not possible to move the penis freely within the preputial sheet. This observation was also recorded. After removing the preputium from the Pars libra penis, each sample was evaluated to see whether lesions and injuries (Figure 1), scars (Figure 2), open wounds due to biting (Figure 3) or severe injuries (Figure 4 and Figure 5) or suppuration (Figure 6) could be detected. 

**Figure 7:**
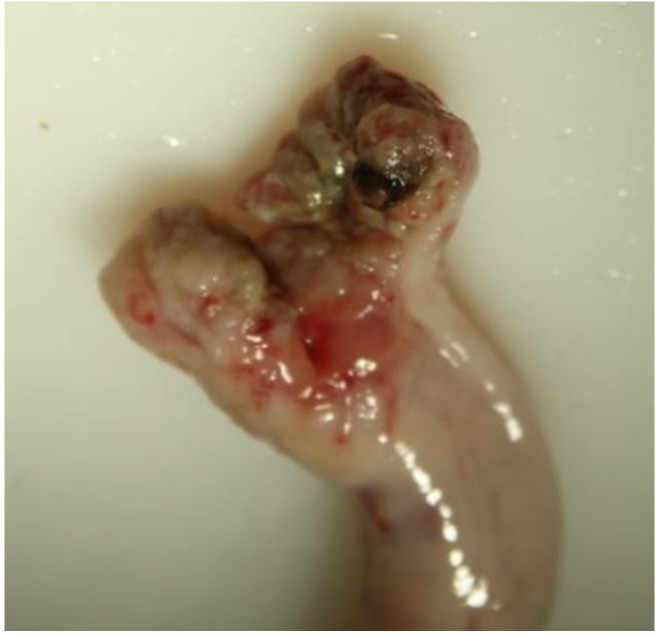
Specimen with partial loss/necrosis (DB).

For the first examinations at the experimental farms, a lesion score was created to summarize the number of major lesions (scars und wounds) on the Glans penis for each animal. Animals were assigned to the following injury score classes (without any = 0, one to three = I, four to six = II, seven to 10 = III, more than 10 = IV) to evaluate the number of lesions (scars und wounds) on the penis. In addition, the size of the major injury per penis and severe injuries (either larger than 1 cm, in combination with suppuration, or losses of a part of a penis) were recorded. Abrasions of the Glans penis, which point to sexual activity but may not be relevant for welfare, were also registered (Figure 7). 

Based on these findings (incidence, lesion type), the evaluation scheme (separate evaluation of the number of scars and wounds, classification of severe injuries) and the sample size (number of animals evaluated per farm) were adapted for the evaluation of samples from commercial farms. For these animals, fresh wounds and scars were counted and the occurrence of severe injuries was recorded and evaluated separately. For comparison with animals from experimental farms, animals from commercial farms were also assigned to the lesion score classes.

All examples shown in Figure 1Figure 2Figure 3Figure 4Figure 5Figure 6 and Figure 7 were derived from the study in domestic boars (experimental and commercial farms), and examples 8 to 10 shown in Figure 8Figure 9 and Figure 10 were from wild boars.

### 2.6. Statistical Analysis

All statistical tests were conducted with SPSS (Version 22.0.0.0). As the number of scars and fresh injuries per animal were not normally distributed, nonparametric tests were used for analysis.

In order to analyze the background for farm differences in the number of scars/animals, an ANOVA was carried to evaluate the effect of age at slaughter, weight at slaughter, number of animals per pen and whether groups were all males or mixed groups. Age and weight at slaughter and the number of animals per group were included as covariates, and the other factors were fixed effects. Effects of genotype were masked by farm effects as a total of eight different genotypes resulting from 11 farms. LS-means were calculated for the overall means and for the effect of mixed *vs.* single-sex group.

## 3. Results

### 3.1. Incidence and Size of Penile Injuries in Experimental Farms

In total, 470 animals were dissected at the slaughterhouse and the penises examined as described above. Penises from barrows (*n* = 85) were small and it was not possible to remove them completely from the preputial sheet without affecting the Glans penis or the Pars libra. These samples were all free of scars, wounds, severe injuries or suppuration. They were not further included into the statistical evaluation. 

In contrast, between 76.6% and 91.3% of the boars had scars or fresh wounds on the penis (see Table 1). The lesion score of penile injuries differed significantly between farms (Kruskal Wallis-test: *p* < 0.001). The analysis of variance revealed no additional influence of age or slaughter weight of the individual. Their assignment to the different injury classes is summarized in Figure 11. The size of the major injury varied between 0.1 and 0.3 cm in 31.5% and was larger than 1 cm in 14.8% of the animals with injured penises (Table 2).

### 3.2. Incidence of Penile Injuries at Commercial Farms

A total of 321 samples were visually evaluated by at least two persons for fresh injuries and scar tissue. The scar tissue was further verified by palpation. The Kruskal-Wallis test revealed no differences between farms in the distribution of fresh injuries, but highly significant differences in the distribution of scars per animal (*p* < 0.001). Similarly, significant median differences between farms could not be established for fresh injuries, but were present for scars (*p <* 0.001). Mean values for animal characteristics and the results of the evaluation are summarized per farm in Table 3.

In order to obtain further information about possible factors influencing the incidence and number of scars, the effects of age and weight at slaughter, number of animals per pen and whether the groups were housed in single-sex or mixed groups were subsequently included into the model of the analysis of variance. Weight at slaughter (df: 1; F = 0.14) and animals per pen (df: 1; F = 2.67) had no significant effect on the number of scars per animal. The age at slaughter (df: 1; F = 21.35; *p* < 0.001) and whether groups were single-sex both revealed a significant (df: 1; F = 10.23; *p* < 0.01) effect on the number of scars. The estimate for age revealed an increase of 0.04/day in the period of 150 to 205 days. The single-sex groups had significantly lower numbers of scars when corrected for the age effect (single-sex: 3.07 + 0.17; mixed: 4.68 + 0.45 number of scars/animal).

### 3.3. Penile Injuries in Wild Boars

**Figure 9:**
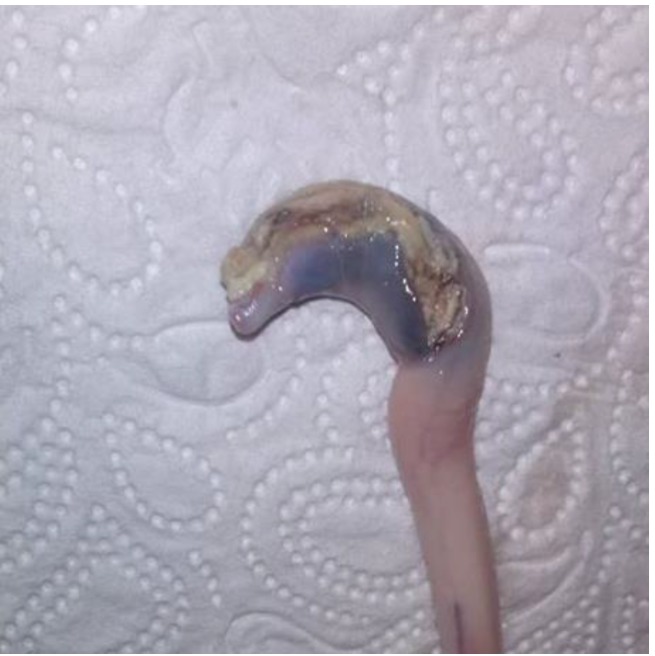
Specimen with severe injury (WB).

**Figure 11:**
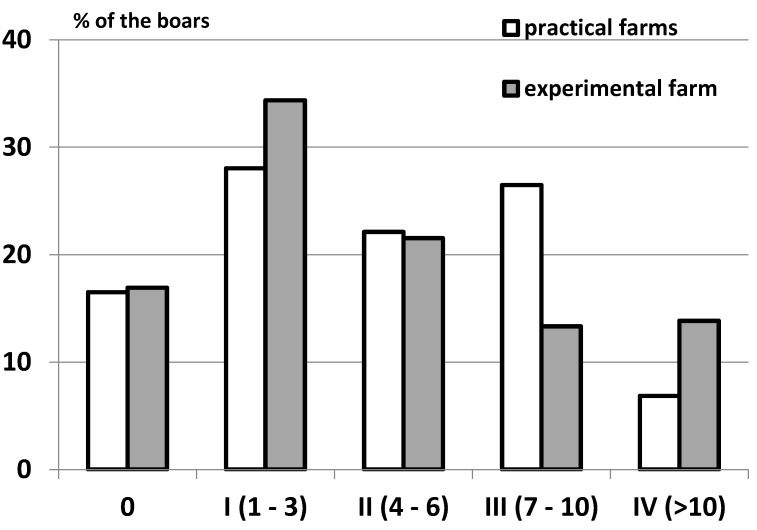
Percentage of boars from commercial (*n* = 321) and experimental farms (*n* = 385) assigned to the different injury score classes 0 to IV (number of wounds and scars).

In six out of the 15 post-pubertal wild boars, penile injuries were observed (40%). Two animals with injuries were younger than one year, two animals were between one and two years of age and two others older than two years. Scars were only found in two animals (one and two scars, respectively), while the number of fresh wounds ranged between one and eight (3.00 ± 2.51 per animal; *n* = 6). Three of the injuries were classified as severe due to extensive suppuration (according to score IV, see Figure 9). The animals with injuries were obtained during the late hunting season between mid-December and mid-January. 

## 4. Discussion

Surgical castration of pigs without pain relief is now considered unacceptable due to welfare reasons. Raising entire males in single-sex groups has been considered an animal-friendly alternative. As a consequence of the increasing concentrations of testicular hormones during the fattening period, entire males are more prone to aggressive and sexual behavior which results in new welfare problems such as stress and injuries [4]. While the occurrence of such problems is well-described in literature, data about the incidence of penis biting and the resulting penile injuries is scarce. On commercial farms, signs of severe injuries have been reported by farmers but were not quantified. Therefore, the incidence of penis injuries in male pigs was studied in experimental farms (first population), commercial farms (second population) and wild boar samples (third population). 

Samples from barrows revealed no injuries and were completely unharmed as the animals were physiologically inhibited to extrude the penis. The separation of penis and prepuce in boars takes place during puberty [12]. In the pre-pubertal wild boars, the penis was similarly attached to the preputium and revealed no injuries at all. In contrast, only 17.7% of the boars from experimental farms examined at the slaughterhouse had unharmed penises. The incidence of penile injuries varied significantly between the three experimental farms with different housing conditions such as space allowance, animals per pen or age and weight at slaughter. The differences between farms in population 1 could not be ascribed to one of these factors. Nevertheless, the majority of the boars revealed multiple scars and fresh wounds of varying sizes and 5.2% of all boars even had severe injuries on the Glans penis. During behavioral observations, boars showed more frequent mounting behavior with extension of the penis, thereby permitting conspecifics to bite at the extended penis. Penis biting, however, was only occasionally recorded during behavioral observation periods and is rarely supported in literature [9,12]. Compared to the observations, the incidence of penile injuries recorded after slaughter by counting wounds and scars was surprisingly high in population 1, but numbers were quite similar to those of boars from commercial farms. 

Differences between farms in the second population were significant and could be ascribed in part to either mixed group *vs.* single-sex housing or to the age at slaughter. The farms with the lowest incidence of injuries slaughtered the boars at a very young age and kept them in single-sex groups. In contrast, a higher incidence was obvious if boars were raised in mixed groups or slaughtered at an older age. Reports have shown that group housing instead of individual housing of boars provokes a higher sexual activity (hyper-sexuality), which can lead to injuries and bleedings of the penis [12]. Regarding the relevance of this phenomenon for animal welfare issues, the very high amount of animals with severe injuries has to be considered. 

The most surprising finding was the incidence of penile injuries in wild boars. Even if the number is very limited the results are valuable for the discussion of the factors leading to penile injuries. Because all wild boars were free-ranging during the hunting season, a limitation of space can be excluded as a cause for penis biting. In fact, injuries were documented in 40% of the post-pubertal animals with half of them being severe injuries. Interestingly, all boars that were hunted before mid-December were neither harmed nor had scars, whereas the animals with fresh wounds and injuries were shot later during the mating season. In wild boars, the main mating season and puberty are triggered by decreasing day length [13,14]. From wild boars it is known that post-pubertal younger boars are inhibited from fertile mating activity during the mating season by the dominant male, and that they stay in bachelor groups not far from the females [15]. Inter-male mounting activity as similarly described for single-sex groups of domestic boars [2,7] has not been reported, but seems likely. 

## 5. Conclusions

The results show a high incidences of penile injuries in boars in commercial as well as in experimental farms. Even in wild boars a considerable proportion of animals with penile injuries was detected during the mating season. Thus, penis biting is a highly relevant and severe welfare problem in boars which is not limited to intensive production systems.

## Figures and Tables

**Figure 8:**
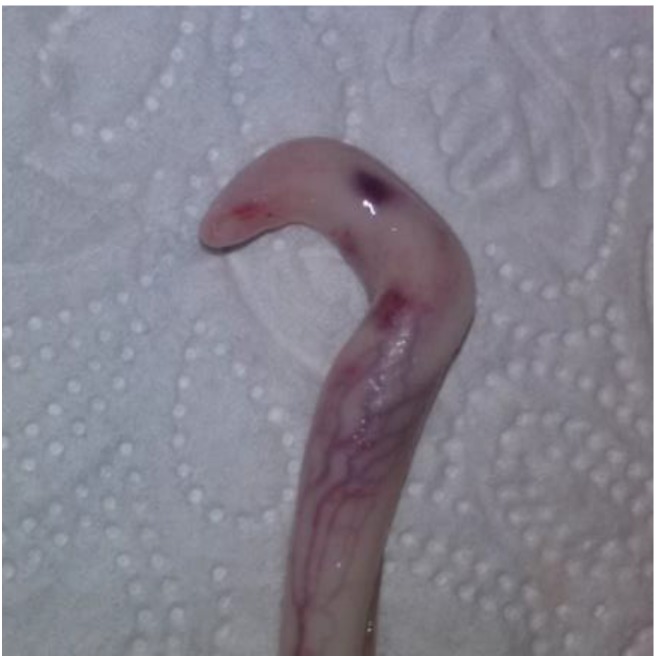
Specimen with hematomas and injuries (WB).

**Figure 10:**
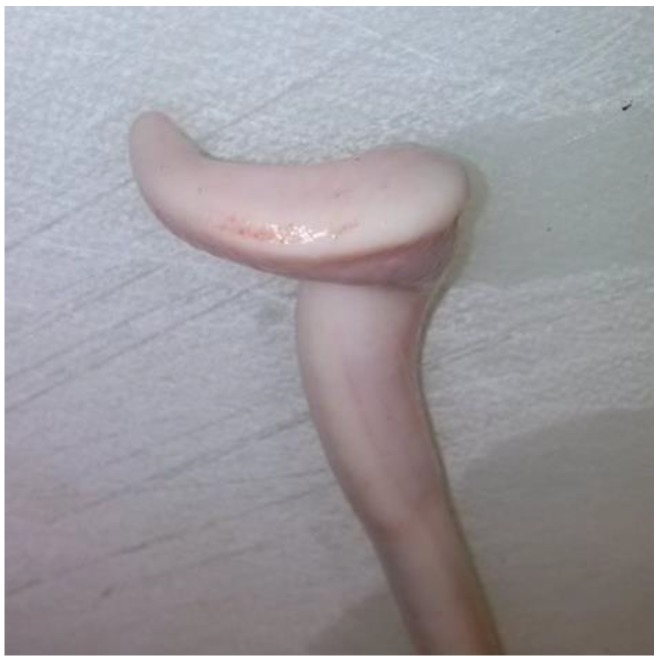
Specimen with thickening of and lesions at the ridge due to sexual activity in a WB.

**Table 1:** Number of boars evaluated (*n*), number of animals per pen, space allowance per animal (m^2^), age (d), slaughter weight (kg) and lesion score of scars and fresh injuries per animal (mean ± SD) evaluated for each farm. (Total: LS-means ± SEM). The percentage of animals with severe injuries and of those without any lesions is also given.

Farm	*n*	Animals/Pen	m^2^/Animal	Age (d)	Slaughter Weight (kg)	Lesion Score/Animal	% Animals with Severe Injuries	% Animals without Lesions
E1	146	21	0.75	195.6 ± 14.4	92.3 + 9.3	2.08 ± 1.36	4.1	13.0
E2	193	24	0.81	190.7 ± 8.8	94.0 ± 7.4	1.39 ± 1.14	5.2	23.3
E3	46	28	1.70	187.0 ± 7.1	98.9 ± 7.3	1.85± 1.19	8.7	8.7
Total	385	2.34	0.89	192.2 ± 0.5751	93.9 ± 0.567	1.70 ± 0.065	5.2	17.7

**Table 2:** Number of boars with penile injuries (total number of boars) and size distribution (%) of the major injury for each experimental farm and in total (mean).

Farm	*n*	% of Animals with Different Sizes (cm) of the Major Injury			
		0.1–0.3	0.4–0.6	0.7–1.0	>1.0
E1	127 (146)	22.8	57.5	15.0	4.7
E2	148 (193)	40.5	41.2	12.2	6.1
E3	42 (46)	26.2	40. 5	23.8	9.5
Total	317 (385)	31.5	47.6	14.8	6.0

**Table 3:** Number of animals evaluated (*n*), number of animals per pen, mean age at slaughter (d), slaughter weight (kg) and the number of scars and fresh injuries per animal (mean ± SD) evaluated for each commercial farm. (Total: LS-means ± SEM). In addition, the percentages of animals with severe injuries and those without any lesions are given for each farm and in total.

Farm	*n*	Animals/Pen	Age (d)	Slaughter Weight (kg)	Number of Scars/Animal	Number of Fresh Wounds/Animal	% Animals with Severe Injuries	% Animals without Lesions
F1	25	42	170	95.5	2.08 ± 2.06	1.08 ± 2.00	4.0	20.0
F2	25	37	205	93.3	3.32 ± 2.23	2.44 ± 2.62	16.0	12.0
F3	24	13	150	92.9	1.32± 1.87	1.24 ± 1.83	4.0	36.0
F4	39	17	200	95	4.10± 2.67	1.51 ± 1.52	25.6	5.1
F5	45	13	205	96	5.18 ± 2.60	1.09 ± 1.18	13.3	6.7
F6	27	10	190	94.8	2.78 ± 2.91	1.26 ± 1.38	7.4	25.9
F7	31	20	197	93.8	2.45 ± 3.10	1.68 ± 2.02	12.9	16.1
F8	30	23	150	93.2	2.87 ± 2.76	1.07 ± 1.53	6.7	20.0
F9	24	35	155	95.5	3.42 ± 2.54	1.54 ± 1.93	4.2	8.3
F10	25	13	155	94.7	3.88 ± 2.83	1.20 ± 1.44	8.0	12.0
F11	25	18	184	91.7	3.52 ± 3.31	1.60 ± 2.18	0.0	32.0
Total	321	21.9	178.3	94.2	3.17 ± 0.150 ^1^	1.43 ± 0.10 ^1^	9.28 ± 6.88%	17.65 ± 9.78%

^1^ SEM.

## References

[1] European Declaration on Alternatives to Surgical Castration of Pigs. http://www.alternativepig.eu/fileadmin/user_upload/PDF/Declaration/castration_pigs_declaration_en.pdf.

[2] Hintze S., Scott D., Turner S.P., Meddle S.L., D’Eath R.B. (2013). Mounting behaviour in finishing pigs: Stable individual differences are not due to dominance or stage of sexual development. Appl. Anim. Behav. Sci..

[3] Fredriksen B., Lium B.M., Marka C.H., Heier B.T., Dahl E., Choinski J.U., Nafstad O. (2006). Entire male pigs in farrow-to-finish pens—Effects on animal welfare. Appl. Anim. Behav. Sci..

[4] Rydhmer L., Hansson M., Lundstrom K., Brunius C., Andersson K. (2013). Welfare of entire male pigs is improved by socialising piglets and keeping intact groups until slaughter. Animal.

[5] Giersing M., Lundström K., Andersson A. (2000). Social effects and boar taint: Significance for production of slaughter boars (*Sus scrofa)*. J. Anim. Sci..

[6] Fredriksen B., Hexeberg C. (2009). The effect of removing animals for slaughter on the behavior of the remaining male and female pigs in the pen. Res. Vet. Sci.,.

[7] Rydmer L., Zamaratskaia G., Andersseon H.K., Algers B., Guillemet R., Lundström K. (2006). Aggressive and sexual behaviour of growing and finishing pigs reared in groups, without castration. Acta Agric. Scand..

[8] Brunius C. (2011). Early Immunocastration of Male Pigs: Effects on Physiology, Performance and Behaviour. Ph.D. Thesis.

[9] Jackson P.G.G., Cockcroft P.D. (2007). Handbook of Pig Medicine.

[10] Isernhagen M., Ritzmann M., Stark J., Zoels S. (2015). The fattening of entire male pigs under conventional housing conditions. Tierarztl Prax.

[11] Isernhagen M. (2014). The Fattening of Entire Male Pigs under Conventional Housing Conditions—Impact on Animal Health and Animal Welfare. Ph.D. Thesis.

[12] Hühn U. (2013). Sex characteristics of young boars during puberty. Nutztierpraxis Aktuell.

[13] Weiler U., Dehnhard M., Hofaecker S., Claus R. (1996). Influence of a light programme on metabolicaly active hormones and food intake in domestic pigs compared to a wild boar. Can. J. Anim. Sci..

[14] Weiler U., Claus R., Louveau I., Schnoebelen-Combes S. (1998). Influence of age and genotype on endocrine parameters and growth performance: A comparative study in Wild boars, Meishan and Large White boars. Livestock Prod..

[15] Mauget R. (1980). Ecological, behavioural and reproductive regulation of wild boars, Sus scrofa, on environmental adaption. Ph.D. Thesis.

